# Infection prevention and control in tertiary care hospitals of Bangladesh: results from WHO infection prevention and control assessment framework (IPCAF)

**DOI:** 10.1186/s13756-022-01161-4

**Published:** 2022-10-06

**Authors:** Md. Golam Dostogir Harun, Md Mahabub Ul Anwar, Shariful Amin Sumon, Md Zakiul Hassan, Tahmidul Haque, Syeda Mah-E-Muneer, Aninda Rahman, Syed Abul Hassan Md Abdullah, Md Saiful Islam, Ashley R. Styczynski, S. Cornelia Kaydos-Daniels

**Affiliations:** 1 Programme for Emerging Infections, Infectious Diseases Division, icddr,b, Dhaka, Bangladesh; 2 Centers for Disease Control and Prevention (CDC), Bangladesh Country Office, Dhaka, Bangladesh; 3 Communicable Disease Control, Directorate General of Health Services, Dhaka, Bangladesh; 4 SafetyNet, Dhaka, Bangladesh; 5 University of New South Wales, Sydney, Australia; 6 Division of Infectious Diseases and Geographic Medicine, Stanford University, Stanford, CA 94305 USA

**Keywords:** Antimicrobial resistance, Facility assessment, Hospital-acquired infection, Infection prevention and control, IPCAF Bangladesh

## Abstract

**Introduction:**

Infection prevention and control (IPC) in healthcare settings is imperative for the safety of patients as well as healthcare providers. To measure current IPC activities, resources, and gaps at the facility level, WHO has developed the Infection Prevention and Control Assessment Framework (IPCAF). This study aimed to assess the existing IPC level of selected tertiary care hospitals in Bangladesh during the COVID-19 pandemic using IPCAF to explore their strengths and deficits.

**Methods:**

Between September and December 2020, we assessed 11 tertiary-care hospitals across Bangladesh. We collected the information from IPC focal person and/or hospital administrator from each hospital using the IPCAF assessment tool.. The score was calculated based on eight core components and was used to categorize the hospitals into four distinct IPC levels– Inadequate, Basic, Intermediate, and Advanced. Key performance metrics were summarized within and between hospitals.

**Results:**

The overall median IPCAF score was 355.0 (IQR: 252.5–397.5) out of 800. The majority (73%) of hospitals scored as ‘Basic’ IPC level, while only 18% of hospitals were categorized as ‘Intermediate’. Most hospitals had IPC guidelines as well as environments, materials and equipments. Although 64% of hospitals had IPC orientation and training program for new employees, only 30% of hospitals had regular IPC training program for the staff. None of the hospitals had an IPC surveillance system with standard surveillance case definitions to track HAIs. Around 90% of hospitals did not have an active IPC monitoring and audit system. Half of the hospitals had inadequate staffing considering the workload. Bed occupancy of one patient per bed in all units was found in 55% of hospitals. About 73% of hospitals had functional hand hygiene stations, but sufficient toilets were available in only 37% of hospitals.

**Conclusion:**

The majority of sampled tertiary care hospitals demonstrate inadequate IPC level to ensure the safety of healthcare workers, patients, and visitors. Quality improvement programs and feedback mechanisms should be implemented to strengthen all IPC core components, particularly IPC surveillance, monitoring, education, and training, to improve healthcare safety and resilience.

## Background

Infection prevention and control (IPC) in healthcare settings is imperative for the safety of patients as well as healthcare providers [1]. IPC remains a cornerstone strategy for preventing hospital-acquired infections (HAI) and antimicrobial resistance (AMR) [2]. HAIs are associated with high rates of morbidity and mortality, affecting over 1.4 million patients annually around the globe and carrying a projected mortality of over 10% [3–5]. Low- and middle-income countries (LMIC) face an undue burden of HAIs with up to 25% of hospitalized patients experiencing HAIs compared to 7% in the high-income countries [6, 7]. These infections heavily strain health systems and incur rising direct and indirect costs [5, 8].

Like many LMICs, IPC practices in Bangladesh have been hindered by overcrowding, understaffing, inadequate environmental cleaning, insufficient hand washing stations, low compliance with recommended hand hygiene practices, poor ventilation, and lack of IPC training [9, 10]. This has resulted in high rates of HAIs. In one study from Bangladesh, 5% of patients hospitalized for > 72 h acquired a hospital-acquired respiratory illness [11]. HAIs can also affect healthcare workers. The same study [11], documented 27% of healthcare workers experienced a respiratory illness during the study period. Another study reported that 2.6% of hospitalized patients and 4% of healthcare workers developed hospital-acquired diarrhea [12]. Other studies from Bangladesh have estimated overall rates of HAIs ranging from 8 to 30%, exacerbated by inadequate IPC [13–15].

The recent COVID-19 pandemic further exposed the gaps in IPC to prevent HAIs. Despite various infection control measures, up to 44% of SARS-CoV-2 infections early in the outbreak were hospital-acquired [16]. In Bangladesh, 9455 frontline health workers have been infected, and 180 physicians died from COVID-19 between March 2020 and December 2021, which indicates substantial deficits in IPC practices [17, 18]. However, throughout the pandemic, hospitals have enhanced IPC efforts leading to fewer hospital-acquired SARS-CoV-2 infections, demonstrating the effectiveness of this strategy against epidemic as well as endemic diseases [19].

Additionally, AMR has been inextricably linked to HAIs in LMIC settings [20]. In 2019, bacterial AMR was directly responsible for around 1.27 million fatalities worldwide and attributable for an estimated 495 million (362–657 million) deaths without infection [21]. AMR is anticipated to result in 9 million excess deaths and a financial loss of $100 trillion in LMICs by 2050 [22]. HAIs and AMR transmission in healthcare settings can be prevented by implementing systematic and effective IPC initiatives [23, 24]. Moreover, IPC is a cost-effective strategy to reduce infections in healthcare settings [19]. Despite substantial progress to minimize HAIs in many parts of the world, several recent events have highlighted the need to improve IPC at both the national and facility levels [25]. In light of this concern, in 2018, WHO released an evidence-based tool on IPC core components titled “The Infection Prevention and Control Assessment Framework (IPCAF)” to assess, analyze, and enhance the IPC activities of a hospital facility [24]. The overall IPCAF score indicates the level of IPC standards of a hospital [1]. Using this framework and the set of tools, healthcare facilities would be enabled to evaluate existing IPC processes and infrastructure and detect relevant problems and shortcomings that require improvement. In Bangladesh, limited systematic evaluations of IPC have been conducted at the national or facility level. This study aimed to assess the current IPC standards and find out the gaps of selected tertiary care hospitals in Bangladesh using the IPCAF tool and identify steps for IPC improvement during the COVID-19 pandemic and beyond.

## Methods and materials

### Study design and study sites

We conducted this cross-sectional survey from September-December 2020. After discussion with the Director-General of Health Services (DHGS), hospital leadership teams, and subject matter experts, we purposively selected 11 tertiary hospitals (bed occupancy ranged from 450 to 2600) across Bangladesh that demonstrated a commitment to enhancing IPC. These hospitals were purposively selected by the government authorities (DGHS) to implement the IPC pilot intervention, which is around 25% of total tertiary care health facilities in Bangladesh, Face-to-face interviews were conducted from respective hospitals with the IPC focal person and/or hospital administrator who had IPC-related expertise.

### Data collection tool—IPCAF

We used the IPCAF tool to assess the current IPC level and resources in selected hospitals. IPCAF is a structured, closed-ended questionnaire with an associated scoring system. This is an established tool to measure IPC activities and identify relevant strengths and weaknesses at acute health care facilities [26]. It comprises eight sections highlighting the eight IPC core components (CC). The results of each question are aggreagated within the eight CCs, with possible scores ranging from 0 to 100 for each CC. The overall IPCAF score was obtained by summing the findings of all eight core components. The eight CCs of the IPCAF questionnaire are as follows:

- CC1: IPC program
- CC2: IPC guidelines
- CC3: IPC education and training
- CC4: HAI surveillance
- CC5: Multi-modal strategies for implementation of IPC interventions
- CC6: Monitoring/audit of IPC practices and feedback
- CC7: Workload, staffing, and bed occupancy
- CC8: Built environment, materials, and equipment for IPC at the facility level

These eight core components include a total of 81 indicators. Based on the overall score obtained, the respective facility was categorized into one of four IPC promotion levels (Table 1).

**Table 1:** IPCAF scoring and Interpretation

IPCAF Score	Category	Interpretation
0–200	Inadequate	IPC core components implementation is deficient. Significant improvement is required
201–400	Basic	Some aspects of the IPC core components are in place, but not sufficiently implemented. Further improvement is required
401–600	Intermediate	Most aspects of the IPC core components are appropriately implemented. The facility should continue to improve the scope, implementation, and quality and focus on the development of long-term plans to sustain and promote the existing IPC program activities
601–800	Advanced	The IPC core components are fully implemented according to the WHO recommendations and appropriate to the facility’s needs

### Data analysis

We completed data entries through MS-Access. For categorical results, descriptive analysis was conducted using a frequency and cross-tabulation analysis. We summarized the indicators by frequency, percentage, and median with interquartile range. Statistical software STATA version 15.0 (STATA Corp Inc., Texas, USA) was used to perform the analyses.

## Results

### Demographic information

All 11 healthcare facilities were tertiary care hospitals, with average bed capacities ranging from 620 to 970. In terms of facility ownership and management, nine of the facilities were public hospitals and two were private hospitals (Table 2). The selected healthcare facilities are also teaching hospitals with huge patient loads. The hospitals have key departments and equip modern healthcare facilities. These are also the referral hospitals, and patients from sub-district and district-level hospitals get admission to these hospitals.

**Table 2:** Demographic information of study hospitals

Facility type	Hospital name	Bed capacity	Bed Capacity (Average)	Annual patient turnover (Average)
Public hospital	Hospital 1	450	970 (450–2600)	85,522
	Hospital 2	450		
	Hospital 3	500		
	Hospital 4	2600		
	Hospital 5	1400		
	Hospital 6	850		
	Hospital 7	500		
	Hospital 8	500		
	Hospital 9	1500		
Private hospital	Hospital 10	580	610 (580–640)	15,500
	Hospital 11	640		

### Distribution of IPCAF score

**Fig. 1:**
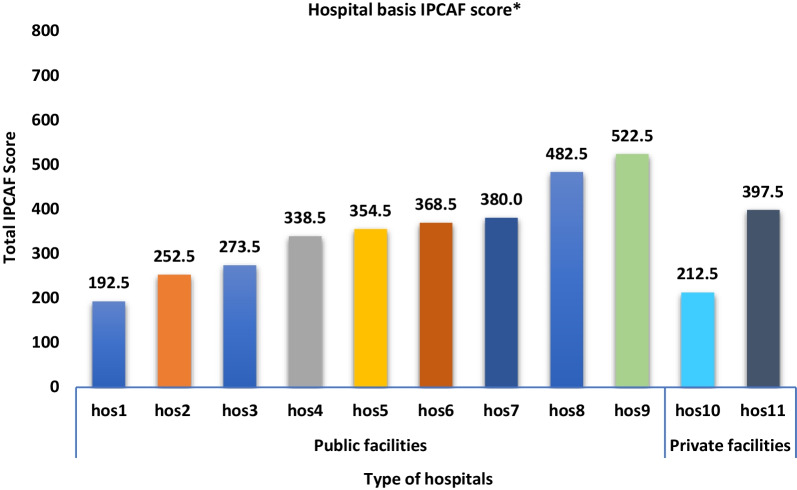
Total IPCAF scores by participating hospitals. *Reference score: 0–200 = Inadequate, 201–400 = Basic, 401–600 = Intermediate, 601–800 = Advanced

The overall median IPCAF score for the participating hospitals was 355.0 (IQR: 252.5–397.5). The two hospitals with the highest IPCAF scores (522.5 and 482.5; Fig. 1) obtained ‘Intermediate’ IPC levels according to the WHO reference range on IPCAF classification. Eight (72.7%) hospitals scored as ‘Basic’ IPC levels, whereas one hospital fell into the ‘Inadequate’ category with a score of 192.5 (Fig. 1).

Based on core component score distribution, the highest median score (67.5) was recorded for both IPC Guidelines (CC2) and Built environment, equipment, and material for IPC (CC8), while HAI Surveillance (CC4) had the lowest score [median 5.0, IQR: 0, − 23.8]. The widest variability was found for IPC guidelines (CC2) and IPC education and training (CC3), and the narrowest for the built environment, equipment, and material for IPC (CC8) (Table 3).

**Table 3:** Distribution of IPCAF score by core component

Core component (CC)	Median (IQR)
CC1: IPC program	50.0 (37.5, 61.3)
CC2: IPC guidelines	67.5 (48.8, 76.3)
CC3: IPC education and training	30.0 (25.0, 57.5)
CC4: HAI surveillance	5.0 (0.00, 23.8)
CC5: Multimodal strategies for implementation of IPC interventions	35.0 (32.5, 52.5)
CC6: Monitoring/audit of IPC practices and feedback	45.0 (26.3, 48.8)
CC7: Workload, staffing and bed occupancy	40.0 (27.5, 50.0)
CC8: Environments, materials and equipment for IPC	67.5 (56.8, 72.3)

### Component-based analysis

#### IPC program (CC1)

The calculated median score for IPC programs was 50.0 (IQR: 37.5–61.3). About three-fourth of hospitals (72.7%) had an IPC program, but only 18.2% hospitals had this program with clearly defined IPC objectives and annual activity plans. The IPC program was supported by a designated professional and/or an IPC committee in less than half of the hospitals (45.5%). Support for IPC goals and indicators (i.e., at executive level meetings, executive rounds, participation in morbidity and mortality meetings) was demonstrable in 36.4% of hospitals. As for microbiological laboratory support, 9 out of 11 hospitals had laboratory capacity for delivering results reliably (timely with sufficient quality), and less than half of the hospitals had IPC program-specific budget allocations (Table 4).

**Table 4:** Key findings of IPCAF assessment in selected tertiary care hospitals of Bangladesh

Core components	Indicators	Frequency (N = 11)	% (n/N) (%)
*CC1: IPC program*			
IPC program	Have an IPC program except for clearly defined objectives	8	72.7
	Program with clearly defined objectives, annual activity plan	2	18.2
	IPC program supported by part-time IPC professional	5	45.5
	All the IPC teams include both doctors and nurses	8	72.7
IPC committee	IPC committee actively supporting the IPC team	5	45.5
	Senior facility leadership represented/ included in the committee	7	63.6
	Senior clinical staff	9	81.8
	Facility management	10	90.9
	Have clearly defined IPC objectives for specific critical areas	9	81.8
Institutional support	Allocated budget specifically for the IPC program	5	45.5
	Demonstrable support for IPC objectives, indicators in the facility	4	36.4
	Have microbiological lab support and deliver results reliably	9	81.8
*CC2: IPC guidelines*			
Available guidelines for	Expertise for developing or adapting guidelines	7	63.6
	Hand hygiene	11	100.0
	Disinfection and sterilization	10	90.9
	Waste management	10	90.9
	Standard precautions	8	72.7
	Healthcare worker protection safety	8	72.7
	Transmission-based precautions	6	54.5
	Prevention of SSI	6	54.5
	Injection safety	5	45.5
	Antibiotic stewardship	1	9.1
Guidelines develop and monitor	Guidelines consistent with national/international guidelines	9	81.8
	Stakeholders developed guidelines on local needs and healthcare workers executed those	7	63.6
	Healthcare workers received specific updated IPC training	5	45.5
	Monitored IPC guideline implementation regularly	5	45.5
*CC3: IPC education and training*			
IPC training	Presence of IPC experts for conduction of training	6	54.5
	Received IPC training during annual new employee orientation	7	63.6
	IPC training not received by healthcare workers	4	36.4
	IPC training not received by cleaners and other supporting staffs	5	45.5
	IPC training not received by Administrative and managerial staff	7	63.6
	No specific IPC training for patients and their family members	9	81.8
Evaluation of IPC training/education	Periodic evaluation of the effectiveness of the IPC training	3	27.3
	Ongoing development/education offered to staff about IPC	3	27.3
*CC4: HAI surveillance*			
Organization of Surveillance	Surveillance is a defined component of IPC programs	0	0
	Trained professionals in basic epi, surveillance and IPC	0	0
	Informatics/IT support to conduct surveillance	0	0
	Personnel responsible for surveillance	1	9.1
Priorities of Surveillance and conducting areas	Prioritization to determine HAIs for surveillance	0	0
	No surveillance for local priority epidemic infections (TB, flu) and vulnerable populations such as neonates, ICU	6	54.5
	Surveillance for:		
	Surgical site infections	2	18.2
	Device associated infections	2	18.2
	Multidrug-resistant colonization	2	18.2
	Impacts on healthcare staff in the clinical, laboratory settings	2	18.2
	Regular evaluate the surveillance	2	18.2
Methods of surveillance	Use of reliable case definitions and standardized data collection methods	0	0
	Not had any processes to regularly review the data quality	10	90.9
	Not had adequate microbiology and lab capacity to support surveillance	6	54.5
	Adequate microbiology and lab capacity to support surveillance through analyzing the antibiotic drug-resistant pattern	2	18.2
Information analysis dissemination, and governance	Not use of surveillance data to develop a tailored plan for improved IPC	10	90.9
	Regular feedback on up-to-date surveillance IPC committee/administration	1	9.1
	Regular feedback on up-to-date surveillance information with doctor/nurse	4	36.4
	Annually feedback on up-to-date surveillance information by written/orally	4	36.4
*CC5: Multi-modal strategies for implementation of IPC interventions*			
Multi-modal element inclusions	Use of multi-modal strategies for implementation of IPC activities	8	72.7
	Education and training: Written or oral or e-learning mode of information	5	45.5
	Safety climate and culture change: Managers/leaders show visible support	3	27.3
	Monitoring and feedback: Monitoring compliance with outcome indicators	6	54.5
	System change: Interventions to ensure the necessary infrastructure and continuous availability of supplies	8	72.7
	Communications and reminders: Reminders, posters, or other advocacy/awareness-raising tools to promote the intervention	9	81.8
Implementation strategy	Strategies include bundles or checklists	0	0
	Regularly link to colleagues from quality improvement and patient safety	5	45.5
	The multidisciplinary team used to implement IPC multimodal strategies	3	27.3
*CC6: Monitoring/audit of IPC training and feedback*			
Monitoring plan	No well-defined monitoring plan with clear goals, targets and activities	10	90.9
	No trained personnel responsible for monitoring/audit of IPC practices	10	90.9
Monitoring indicators	Transmission-based precautions and isolation	4	36.4
	Usage of alcohol-based hand rub or soap	6	54.5
	Wound dressing change	7	63.6
	Hand hygiene compliance	8	72.7
	Cleaning of the ward environment	9	81.8
	Disinfection and sterilization	9	81.8
	Consumption/usage of antimicrobial agents	4	36.4
Feedback and auditing report	Provide feedback on IPC performance audit report	0	0
	Conduct WHO hand hygiene self-assessment survey	2	18.2
	Reporting of monitoring data annually and assess safety cultural factors	1	9.1
*CC7: Workload, bed staffing and occupancy*			
Staffing	Staffing level assessment in the facility	3	27.3
	System of staffing needs assessments during staffing levels deemed to low	5	45.5
	Maintenance of WHO/national said ratio for Health care worker (HCW) to patients in around 50 of total units	6	54.5
Bed occupancy	Facility’s ward design in accordance with international standards only in certain departments	5	45.5
	Bed occupancy for one patient per bed for all units (including emergency departments and pediatrics)	6	54.5
	Patients NOT placed in beds standing in the corridor outside of the room	5	45.5
	adequate spacing of > 1 m between patient beds for all units (including emergency departments and pediatrics)	3	27.3
	No system to assess and respond when adequate bed capacity is exceeded	4	36.4
*CC8: Built environment, materials and equipment for IPC at the facility level*			
Water	Water services are available at all times and of sufficient quantity	10	90.9
	Reliable safe drinking water station present and accessible at all times	7	63.6
Hand hygiene, sanitation	Functional hand hygiene station with reliably available supplies	8	72.7
	Functional and sufficient number (≥ 4) toilets/improved latrines available	4	36.4
Power supply, ventilation	Functional environmental ventilation available in patient-care areas	11	100
	Sufficient energy/power supply available day and night for all uses	8	72.7
	Appropriate and well-maintained materials for cleaning are available	7	63.6
Cohorting and PPE use	Sufficient and continued availability of PPE for HCW	6	54.5
	Single room is available for cohorting	2	18.2
	Suitable room is available (except a single room) for patient cohorting	6	54.5
Medical waste and sewage management	Functional waste collection containers to all waste generation points	7	63.6
	Functional burial pit/fenced waste dump or municipal pick-up available	6	54.5
	Functional incinerator or alternative treatment technology available	1	9.1
	Functional wastewater treatment system available	2	18.2
Decontamination and sterilization	Functioning reliably dedicated decontamination area/ sterile department	6	54.5
	Reliably have sufficient sterile and disinfected equipment for everyday use	9	81.8
	Disposable items are continuously available when necessary	11	100

#### IPC guidelines (CC2)

CC2 had the highest median score at 67.5 (IQR: 48.8–76.3). Hand hygiene guidelines were available in all hospitals while guidelines for disinfection, sterilization, and waste management were found in 90.9% of hospitals. Only 9.1% of hospitals had antibiotic stewardship guidelines. Around half of the hospitals (54.5%) had guidelines for transmission-based precautions, surgical site infection prevention, and injection safety. Where these guidelines were available, they were consistent with national/international guidelines in most of the hospitals (81.8%). For IPC activities, 63.6% of hospitals had the expertise for developing or adapting IPC guidelines. In 45.5% of hospitals, healthcare providers received specific training on updated IPC guidelines, including regular monitoring systems for guideline implementation (Table 4).

#### IPC education and training (CC3)

IPC education and training varied highly among the study hospitals (median score 30.0, IQR: 25.0–57.5). IPC experts for training were found in more than half of the hospitals (54.5%), though healthcare workers in a significant number of hospitals (36.4%) never or rarely received any IPC training. The cleaners and administrative staff did not participate in any training on IPC in 45.5% and 63.6% of hospitals, respectively. Only 18.2% of hospitals had arrangements for specific IPC training for patients and their family members (Table 4).

#### HAI surveillance (CC4)

HAI surveillance received the lowest score in the facility assessment, with a median of 5.0 (IQR: 0–23.8). None of the hospitals had a surveillance system as a defined component of IPC, nor did they use a standardized case definition for surveillance of HAIs. All hospitals cited a lack of necessary IT support and specialized professionals skilled in epidemiology to carry out surveillance. Only two hospitals conducted regular analysis for antimicrobial drug resistance patterns (Table 4).

#### Multi-modal strategies (CC5)

The majority of hospitals (72.7%) were found to use multi-modal strategies for the implementation of IPC activities and the median value for CC5 recorded 35.0 (IQR: 32.5–52.5). More than half of the hospitals (54.5%) had monitoring compliance maintained with outcome indicators. Managers showed visible support only in few hospitals (27.3%) for safety climate and culture change. In all hospitals except one, reminders, posters, or awareness-raising tools were used to promote interventions. However, less than one-third of hospitals (27.3%) had multi-disciplinary teams for the implementation of various strategies (Table 4).

#### Monitoring and audit of IPC practices and feedback (CC6)

CC6 had a median value of 45.0 (IQR: 26.3–48.8), which indicates a lack of monitoring and audit practices in study hospitals. In the majority of hospitals (90.9%), monitoring plans with clear goals, targets, or activities, including trained monitoring persons were absent. The most monitored indicators were disinfection and sterilization of medical equipment and cleaning of ward environments (81.8%) followed by hand hygiene compliance (72.7%) and wound dressing changes (63.6%). Only two hospitals conducted the WHO hand hygiene self-assessment survey, of which one hospital had annual reporting of monitoring data (Table 4).

#### Workload, staffing, and bed occupancy (CC7)

The median score for workload, staffing, and bed occupancy was 40.0 (IQR: 27.5–50.0). Staffing level assessments were found in only 27.3% of hospitals. Around 55% of hospitals maintained the WHO/national proposed ratio for healthcare workers to patients in around half of their total units. More than half of the hospitals (54.5%) had availability of one patient per bed for all units. Less than one-third hospitals (27.3%) maintained adequate spacing (> 1 m) between patient beds for all units (Table 4).

#### Built environment, materials, and equipment for IPC (CC8)

The built environment, materials, and equipment were frequently conducive to maintaining IPC with a median score of 67.5 (IQR: 56.8–72.3). Around 90.9% of hospitals had available water services with sufficient quantity, and reliable and accessible safe drinking stations were found in 63.6% of hospitals. Regarding ventilation systems, all the hospitals had functional environmental ventilation (natural or mechanical) available in patient-care areas. Functional hand hygiene stations with reliably accessible supplies of alcohol-based hand-rub solution or soap and single-use towels were present at the majority of the hospitals (72.7%). Single room for cohorting was available only in 18.2% of healthcare settings, and over half of the hospitals maintained a sufficient and continuous PPE supply. Functional waste collection containers were found at waste generating points in 63.6% of settings, but only two hospitals had functional incinerator or wastewater treatment systems. More than 88% of hospitals possessed sufficient reliable equipment that was sterile and disinfected for daily usage (Table 4).

## Discussion

We assessed the existing IPC level of selected tertiary care hospitals in Bangladesh using the WHO IPCAF tool. There are very few studies conducted in South Asian countries, including Bangladesh, using the IPCAF tool to assess the IPC condition in tertiary care hospitals. This assessment has provided us with valuable insights into the actual scenario of key IPC structures, implementation, and processes in these healthcare settings. Findings from other studies have revealed that adherence to proper IPC measures can reduce the incidence of HAIs by up to 70% in healthcare settings [27]. This assessment revealed that most facilities only met a basic IPC level [28] which is consistent with a recently conducted global study [1]. While some aspects (IPC guidelines, IPC environment, material, equipment, IPC program) of the IPC core components were frequently in place, not all were sufficiently implemented, indicating that substantial improvement is required.

Although it was not designed in response to COVID-19, the co-occurrence of the pandemic underscored the importance of IPC and the need to identify opportunities for improvement.PPE deficits may have been exacerbated by the tremendous global supply chain gaps that emerged as the demand for PPE in response to SARS-CoV-2 grew. Regardless, the findings from this study identify ongoing crucial IPC deficiencies prevailing at tertiary hospitals that likely contributed to nosocomial cases of SARS-CoV-2. The resilience of healthcare facilities in containing and treating disease without propagating infectious threats will depend on enhancing IPC practices in response to regular assessments such as was conducted in this study.

Two hospitals attained intermediate IPC levels. These facilities were remarkable for obtaining higher scores in most of the indicators compared to other hospitals. Only one facility ranked at the inadequate IPC level. No facility achieved an advanced IPC level. Therefore, we observed a profound degree of heterogeneity through this facility-level IPC assessment. These results of IPC capacityare similar to other facility assessments conducted in South Asia and other middle-income countries [29] and are concerning.

Regarding the IPCAF scores, marked differences occurred among IPCAF core components between the tertiary care hospitals. IPC programs (CC1) were frequently present, though the deliverables of these programs are less clear given the lack of defined objectives and annual activity plans. This highlights the need for strategically delineating the deliverables of an IPC program to ensure its impact. Other studies also have testified that an IPC program’s success depends on clear communications and the specification of objectives [30]. Additionally, less than half of the hospitals had an IPC team comprising of dedicated IPC professionals to support the IPC program; all the designated individuals were temporarily appointed. This reveals an inadequate institutional commitment to strengthening IPC, which is supported by the fact that less than half of the facilities had an allocated budget for conducting IPC activities. Similar deficits in budget allocations have been observed in a global situational analysis, revealing this to be a widely pervasive challenge [31]. There always has been a direct relationship between the availability of funds and increased IPC preparedness. An IPC programme with dedicated team and governance, is crucial to reduce the spread of infectious diseases in the hospital setting [32]. Lack of allocated money, lack of clear and specific objectives to run the program, and lack of dedicated IPC teams demonstrate the need for greater IPC prioritization. In collaboration with Directorate General of Health Services (DGHS), the hospital authorities can enhance the institutional commitment through contextualized IPC programs with priority-based objectives and designated teams.

Although IPC guidelines (CC2) were commonly available, antibiotic stewardship guidelines were found only in one hospital. This might be a contributing factor to physicians’ lack of knowledge towards rational prescribing of antibiotics [33]. The establishment of an antibiotic stewardship program (ASP) could be an essential step in educating clinicians to optimize the use of antibiotics for effective treatment of infections and protect patients from antibiotic resistance [34]. IPC training and education (CC3) were less frequent and sometimes absent, despite being six months into the COVID-19 pandemic. A study from Bangladesh also highlighted that healthcare workers were not receiving quality and sufficient training around IPC, with 85% of participants reporting they received no formal training on infection control [35]. Regular IPC training was also found to be lacking from studies in Ghana, Pakistan, and even Austria [29, 36, 37]. These findings demonstrate that the importance of regular IPC training may not be well understood by hospital administration or that IPC experts to facilitate such training are lacking. Cleaners, who are a core part of hospital staff and significantly contribute to maintaining hospital hygiene, were found to receive IPC training even less frequently than healthcare workers in our study. This finding is consistent with a study conducted in Turkey where about 57% of the cleaners did not receive any formal training on infection control [38]. The hospitals should regularly arrange motivation sessions and hands-on IPC training for all the staff, particularly for cleaners.

HAI surveillance (CC4) was found to be routinely lacking, stemming from inadequate microbiology and lab capacity, lack of IT support, and absence of experts trained in basic epidemiology. HAI surveillance cannot be effectively conducted if each of these parameters is not in place. Hospital-based surveillance allows the estimation of local burden and incidence of HAI so that appropriate IPC measures can be taken [4, 39]. Given the known burden of antimicrobial drug resistance in admitted patients with HAIs [40], such a scenario ultimately puts the patients at risk of receiving inappropriate care. HAI surveillance should be improved by concurrent building capacity in laboratory diagnostics and epidemiologic methods.

Multi-modal strategies (CC5) is a comparatively new concept that is gradually becoming more prevalent in IPC practice [41–43]. Most of the hospitals achieved a low score for this component, which indicates the lack of awareness about the need to have a multi-disciplinary team to provide inputs on and implement a variety of strategies. The low utilization of multi-modal strategies may arise because of a lack of interdepartmental cooperation. Studies conducted in Pakistan, Austria, and Germany also reported similar findings, although the last two countries were in high-income settings and technologically more advanced than Bangladesh [29, 36, 44].

Study facilities varied in monitoring and auditing of IPC practices (CC6) as well as workload, staffing, and bed occupancy levels (CC7). A well-defined monitoring plan with proper goals, targets, and activities was absent in all hospitals, except one public facility with a strong enforcement culture and greater structural and logistical support. None of the facilities provided regular feedback reports on IPC performance audits to any upper administration, which shows limited institutional investment in making improvements around IPC. Gilbert and Kerridge asserted that poor leadership often has a deep connection with a lack of proper IPC practices in the facility and negatively impacts the overall compliance rate [45]. Nearly half of facilities demonstrated understaffing and overcrowding, which have been demonstrated to be significant risk factors for HAIs in many previous studies [46, 47]. Although the system may have been unusually burdened by surges of COVID-19, previous studies from Bangladesh have demonstrated similar rates of deficiencies on these metrics [10]. Overcrowding and inadequate staffing in these hospitals reveal another opportunity for improvement in IPC practices but require high-level institutional support and planning. The policymakers should fill up vacancies on a priority basis and foster the recruitment process until a standard patient to staff ratio is obtained.

The last core component (CC8) assesses the infrastructure, materials, and equipment for optimum IPC practices in a healthcare setting. Data showed that the hospitals' water, electricity, light, and ventilation (mechanical or natural) system was generally satisfactory as more than 70% of the facilities had these amenities in abundance, which is consistent with Bangladesh’s status as a lower-middle-income country. However, some of the institutions were found to be lacking a sufficient number of functional toilets and hand hygiene stations with regular supplies of soap and hand rub solution. This may have resulted from decreased access to maintenance and repair services during the pandemic as service technicians may have been concerned about exposure to SARS-CoV-2 in healthcare facilities. The lack of functional hygiene stations can lead to poor compliance with hand hygiene among healthcare workers, which is in line with a study’s findings on hygiene practices in Bangladesh (48). Additionally, there was no validation of the adequacy of ventilation, which may have contributed to SARS-CoV-2 transmission. About half of the facilities were not able to provide an adequate amount of PPE for protecting the healthcare workers. A regular supply of sufficient PPE is crucially important both for the safety of patients and staff and also to interrupt disease transmission. The existing scenario may have been exacerbated by disruptions to PPE supply chains that were caused by the pandemic but also placed healthcare workers at heightened occupational risk. Availability of cleaning materials may be one of the simplest and least expensive ways to improve IPC, though these were far from universally available, posing an ongoing threat to the spread of HAIs. The hospital authorities should regularly track and maintain an PPE supply and ensure the optimum use of PPE through monitoring.

### Strengths and limitations

The study provided us with an opportunity to capitalize the IPCAF tool at an expanded range to assess the present IPC situation and identify critical gaps towards IPC implementation in tertiary hospitals. These findings can be used to help policymakers increase investment in and prioritization of IPC to ensure the safety of healthcare workers in Bangladesh during the COVID-19 pandemic and beyond.

A limitation of this study is that it was carried out in selected tertiary hospitals of Bangladesh which does not provide a nationwide representative result. Incorporating hospitals in different regions that offer varying levels of care could have revealed greater heterogeneity in existing IPC level. Furthermore, many of the indicators were based on self report by a designated IPC person. The assessments could be made more robust with objective data to support their determinations. However, this assessment was carried out in late 2020 when the entire world was experiencing a global pandemic. Because the pandemic generated heightened awareness around IPC, the findings from this assessment may be elevated compared with normal time.

## Conclusion

By revealing the current state of IPC preparedness and shortcomings in tertiary care hospitals, this study can provide a useful framework for policymakers to not only assess the current scenario but also to design strategic improvement plans. This study demonstrates clear areas of need that could benefit from enhanced commitment and stakeholder engagement. The data suggests that the area in most need of improvement is CC4 HAI Surveillance as it is the core components for some of the other CCs. The higher scores in other areas suggest the need to improve assessment methodology as it is problematic to have high scores without adequate CC4 HAI Surveillance data. For establishing an effective IPC system, national IPC standards have to be upgraded first with context-specific training and close monitoring of collected data. Participants from all designated disciplines should collaboratively design and champion a diverse set of approaches to make IPC programs a success.

## Data Availability

The authors are responsible for the data described in this manuscript. The dataset generated and analyzed are available from the corresponding author upon request and some data portions are enclosed within the annex section.

## Abbreviations

AMR: Anti-microbial resistance
CC: Core component
DGHS: Directorate general of health services
HAI: Hospital-acquired infection
IPC: Infection prevention and control
IPCAF: Infection prevention and control assessment framework
IRB: Institutional review board
IT: Information and technology
LMIC: Low and middle-income country
WHO: World Health Organization

## References

[1] Tomczyk S, Twyman A, de Kraker ME, Rehse APC, Tartari E, Toledo JP (2022). The first WHO global survey on infection prevention and control in health-care facilities. Lancet Infect Dis.

[2] Chua AQ, Verma M, Hsu LY, Legido-Quigley H (2021). An analysis of national action plans on antimicrobial resistance in Southeast Asia using a governance framework approach. Lancet Reg Health-Western Pacific.

[3] Allegranzi B, Pittet D (2008). Preventing infections acquired during health-care delivery. Lancet.

[4] World Health Organization. Report on the burden of endemic health care-associated infection worldwide. 2011.

[5] Allegranzi B, Nejad SB, Combescure C, Graafmans W, Attar H, Donaldson L (2011). Burden of endemic health-care-associated infection in developing countries: systematic review and meta-analysis. Lancet.

[6] Pittet D, Allegranzi B, Sax H, Dharan S, Pessoa-Silva CL, Donaldson L (2006). Evidence-based model for hand transmission during patient care and the role of improved practices. Lancet Infect Dis.

[7] Bardossy AC, Zervos J, Zervos M (2016). Preventing hospital-acquired infections in low-income and middle-income countries: impact, gaps, and opportunities. Infect Dis Clin.

[8] Tess B, Glenister H, Rodrigues L, Wagner M (1993). Incidence of hospital-acquired infection and length of hospital stay. Eur J Clin Microbiol Infect Dis.

[9] Shahida S, Islam A, Dey B, Islam F, Venkatesh K, Goodman A (2016). Hospital acquired infections in low and middle income countries: root cause analysis and the development of infection control practices in Bangladesh. Open J Obstet Gynecol.

[10] Rimi NA, Sultana R, Luby SP, Islam MS, Uddin M, Hossain MJ (2014). Infrastructure and contamination of the physical environment in three Bangladeshi hospitals: putting infection control into context. PLoS One.

[11] Gurley ES, Zaman RU, Sultana R, Bell M, Fry AM, Srinivasan A (2010). Rates of hospital-acquired respiratory illness in Bangladeshi tertiary care hospitals: results from a low-cost pilot surveillance strategy. Clin Infect Dis.

[12] Bhuiyan MU, Luby SP, Zaman RU, Rahman MW, Sharker MY, Hossain MJ (2014). Incidence of and risk factors for hospital-acquired diarrhea in three tertiary care public hospitals in Bangladesh. Am J Trop Med Hyg.

[13] Sutradhar KB, Saha A, Huda NH, Uddin R (2014). Irrational use of antibiotics and antibiotic resistance in southern rural Bangladesh: perspectives from both the physicians and patients. Annu Res Rev Biol.

[14] Amin ZA, Nahar N (2017). Hospital acquired infection in a tertiary military hospital in Dhaka, Bangladesh. Int J Infect Dis Therapy.

[15] Miah KA, Chowdhury MZ, Johora FT, Khatun S (2019). Causative organisms of hospital acquired infections among the pediatric patients in tertiary level hospitals of Dhaka city. Update Dental College J.

[16] Barranco R, Vallega Bernucci Du, Tremoul L, Ventura F (2021). Hospital-acquired SARS-Cov-2 infections in patients: inevitable conditions or medical malpractice?. Int J Environ Res Public Health.

[17] MH O. Over 180 doctors killed due to coronavirus in Bangladesh. Dhaka Tribune. 2021.

[18] Association BM. Total affected (Doctors, Nurses & Health workers); https://bma.org.bd/covid-19/Total%20Affected%20Doctor,%20Nurse%20&%20Staff.pdf. 2021.

[19] Breathnach AS (2013). Nosocomial infections and infection control. Medicine.

[20] Laxminarayan R, Duse A, Wattal C, Zaidi AK, Wertheim HF, Sumpradit N (2013). Antibiotic resistance—the need for global solutions. Lancet Infect Dis.

[21] Murray CJ, Ikuta KS, Sharara F, Swetschinski L, Aguilar GR, Gray A (2022). Global burden of bacterial antimicrobial resistance in 2019 a systematic analysis. Lancet.

[22] O'Neill J. Tackling drug-resistant infections globally: final report and recommendations. 2016.

[23] Storr J, Kilpatrick C, Allegranzi B, Syed SB (2016). Redefining infection prevention and control in the new era of quality universal health coverage. J Res Nurs.

[24] Storr J, Twyman A, Zingg W, Damani N, Kilpatrick C, Reilly J (2017). Core components for effective infection prevention and control programmes: new WHO evidence-based recommendations. Antimicrob Resist Infect Control.

[25] Mendelson M, Matsoso MP (2015). The World Health Organization global action plan for antimicrobial resistance. SAMJ South African Med J.

[26] World Health Organization. Infection prevention and control assessment framework at the facility level. World Health Organization. 2018.

[27] Evans S, Agnew E, Vynnycky E, Stimson J, Bhattacharya A, Rooney C (2021). The impact of testing and infection prevention and control strategies on within-hospital transmission dynamics of COVID-19 in English hospitals. Philos Trans R Soc B.

[28] Organization WH. Infection prevention and control assessment framework at the facility level. World Health Organization. 2018.

[29] Savul S, Lalani FK, Ikram A, Khan MA, Khan MA, Ansari J (2020). Infection prevention and control situation in public hospitals of Islamabad. J Inf Develop Ctries.

[30] Houghton C, Meskell P, Delaney H, Smalle M, Glenton C, Booth A (2020). Barriers and facilitators to healthcare workers’ adherence with infection prevention and control (IPC) guidelines for respiratory infectious diseases: a rapid qualitative evidence synthesis. Cochrane Database Syst Rev.

[31] Tartari E, Tomczyk S, Pires D, Zayed B, Rehse AC, Kariyo P (2021). Implementation of the infection prevention and control core components at the national level: a global situational analysis. J Hosp Infect.

[32] Jeong Y, Joo H, Bahk H, Koo H, Lee H, Kim K. Implementation of infection prevention and control components in 1442 hospitals in the Republic of Korea: evaluation using the WHO Infection Prevention and Control Assessment Framework (IPCAF). 2022. 10.1186/s13756-022-01107-w PMC9101985 35562838

[33] Sumon SA, Islam S, Harun GD (2022). Perceptions toward and practices regarding antibiotic stewardship and use among physicians at tertiary-care public hospitals in Bangladesh. Antimicrob Steward Healthcare Epidemiol.

[34] Baroudi R, Flaugher M, Grace E, Zakria D (2015). The importance of an antimicrobial stewardship program. Fed Pract.

[35] Sumon MSA, Parveen S, Hassan MZ, Babar MRK, Chanda KF, Rahman M, editors. 866. Assessment of Infection Control Training among Healthcare Workers in Three Tertiary Care Public Hospitals, Bangladesh, 2015–17. Open Forum Infectious Diseases; 2020: Oxford University Press.

[36] Aghdassi SJS, Grisold A, Wechsler-Fördös A, Hansen S, Bischoff P, Behnke M (2020). Evaluating infection prevention and control programs in Austrian acute care hospitals using the WHO Infection prevention and control assessment framework. Antimicrob Resist Infect Control.

[37] Oppong TB, Amponsem-Boateng C, Kyere EKD, Wang Y, Gheisari Z, Oppong EE (2020). Infection prevention and control preparedness level and associated determinants in 56 acute healthcare facilities in Ghana. Infect Drug Resist.

[38] Demirturk N, Demirdal T (2006). Effect of a training program for hospital cleaning staff on prevention of hospital-acquired infection. Infect Control Hosp Epidemiol.

[39] World Health Organization. Worldwide country situation analysis: response to antimicrobial resistance. World Health Organization Geneva; 2015.

[40] Cassini A, Högberg LD, Plachouras D, Quattrocchi A, Hoxha A, Simonsen GS (2019). Attributable deaths and disability-adjusted life-years caused by infections with antibiotic-resistant bacteria in the EU and the European Economic Area in 2015: a population-level modelling analysis. Lancet Infect Dis.

[41] Allegranzi B, Sax H, Bengaly L, Riebet H, Minta DK, Chraiti M-N (2010). Successful implementation of the World Health Organization hand hygiene improvement strategy in a referral hospital in Mali. Africa Infect Control Hospital Epidemiol.

[42] Allegranzi B, Gayet-Ageron A, Damani N, Bengaly L, McLaws M-L, Moro M-L (2013). Global implementation of WHO's multimodal strategy for improvement of hand hygiene: a quasi-experimental study. Lancet Infect Dis.

[43] Zingg W, Cartier V, Inan C, Touveneau S, Theriault M, Gayet-Ageron A (2014). Hospital-wide multidisciplinary, multimodal intervention programme to reduce central venous catheter-associated bloodstream infection. PLoS One.

[44] Aghdassi SJS, Hansen S, Bischoff P, Behnke M, Gastmeier P (2019). A national survey on the implementation of key infection prevention and control structures in German hospitals: results from 736 hospitals conducting the WHO infection prevention and control assessment framework (IPCAF). Antimicrob Resist Infect Control.

[45] Gilbert GL, Kerridge I (2019). The politics and ethics of hospital infection prevention and control: a qualitative case study of senior clinicians’ perceptions of professional and cultural factors that influence doctors’ attitudes and practices in a large Australian hospital. BMC Health Serv Res.

[46] Schwab F, Meyer E, Geffers C, Gastmeier P (2012). Understaffing, overcrowding, inappropriate nurse: ventilated patient ratio and nosocomial infections: which parameter is the best reflection of deficits?. J Hosp Infect.

[47] Manojlovich M, Sidani S, Covell CL, Antonakos CL (2011). Nurse dose: linking staffing variables to adverse patient outcomes. Nurs Res.

[48] Harun MGD, Sumon SA, Mohona TM, Hassan MZ, Rahman A, Abdullah SAHM (2022). Compliance and constraints of hand hygiene among healthcare workers in Bangladesh. Antimicrob Stewardship Healthcare Epidemiol.

